# Phylogenetic detection of conserved gene clusters in microbial genomes

**DOI:** 10.1186/1471-2105-6-243

**Published:** 2005-10-03

**Authors:** Yu Zheng, Brian P Anton, Richard J Roberts, Simon Kasif

**Affiliations:** 1 Bioinformatics Graduate Program, Boston University, Boston, MA, USA; 2 New England Biolabs, Beverly, MA, USA; 3 Department of Biomedical Engineering, Boston University, Boston, MA, USA; 4 Center for Advanced Genomic Technology, Boston University, Boston, MA, USA

## Abstract

**Background:**

Microbial genomes contain an abundance of genes with conserved proximity forming clusters on the chromosome. However, the conservation can be a result of many factors such as vertical inheritance, or functional selection. Thus, identification of conserved gene clusters that are under functional selection provides an effective channel for gene annotation, microarray screening, and pathway reconstruction. The problem of devising a robust method to identify these conserved gene clusters and to evaluate the significance of the conservation in multiple genomes has a number of implications for comparative, evolutionary and functional genomics as well as synthetic biology.

**Results:**

In this paper we describe a new method for detecting conserved gene clusters that incorporates the information captured by a genome phylogenetic tree. We show that our method can overcome the common problem of overestimation of significance due to the bias in the genome database and thereby achieve better accuracy when detecting functionally connected gene clusters. Our results can be accessed at database GeneChords .

**Conclusion:**

The methodology described in this paper gives a scalable framework for discovering conserved gene clusters in microbial genomes. It serves as a platform for many other functional genomic analyses in microorganisms, such as operon prediction, regulatory site prediction, functional annotation of genes, evolutionary origin and development of gene clusters.

## Background

In microorganisms, it is often seen that genes tend to locate in conserved proximity in a number of genomes forming conserved gene clusters [1]. Further analyses often uncover biologically meaningful relationships between genes with conserved proximity [1-3]: they are often co-transcribed as operons [4], or co-regulated as part of a larger biochemical network [5-7]. Examples include the widely present DNA restriction and modification gene pairs [8], which provide a way of defending against bacteriophage and other foriegn DNA, and the two component systems which respond to changes in environmental conditions [9]. Thus, delineation of conserved gene clusters will help reveal functional relationships between genes within them [10]. In addition, the conserved gene clusters provide an invaluable resource for corroborating the growing number of co-expressed gene sets obtained from microarray based mRNA expression experiments.

The increasing accumulation of genome sequence data has facilitated the practice of finding conserved gene clusters since both similarity and synteny information can be easily obtained [11]. Various computational methods for finding clusters have been described [1,6,12-16]. An important issue has been the estimation of significance of the observed conserved proximity. It is generally accepted that phylogenetic distances between genomes largely determine the significance: conservation is deemed more significant when a cluster appears in distantly related genomes than in closely related ones. The rationale is that the greater the length of time a gene cluster has persisted, the more it has resisted dissolution by recombination events, and the stronger the selective pressure to maintain it. Previous published methods have incorporated phylogenetic information into the estimation of significance by using either 16S RNA distance [1] or statistical methods [12]. However, few efforts have focused on the development of a full evolutionary model to describe conserved gene clusters. Empirical approaches include grouping closely related genome into clades [7] or choosing a subset of genomes based on knowledge of the evolution of microorganisms [14]. Although the latter approaches are efficient in finding non-trivial conserved gene clusters, they do not scale automatically, which is problematic with more and more sequenced genomes.

Moreover, sequenced organisms are often close relatives of known model organisms or pathogens selected for biomedical reasons, so phylogenetic coverage can be rather sparse and biased. For example, there are many more close relatives of *Escherichia coli *than those of *Helicobacter pylori *in the genome database. Thus, simply counting the number of genomes where a gene cluster is conserved often overestimates the significance of conservation of a cluster observed in microorganisms such as *E. coli*.

Delineation of evolutionarily conserved gene clusters must take into account a stochastic model of evolution of these clusters. In this paper we used a simplified stochastic generative evolutionary process where an organism inherits a gene cluster from its immediate ancestor with a probability proportional to the evolutionary distance in the tree. This model facilitates an efficient computation of the probability that a particular pattern of conservation in many genomes is observed. In particular, observing a large number of occurrences in closely related genomes will not carry the same significance as the occurrence of a cluster in a wider evolutionary phylum.

We define a tree-based probabilistic conservation score and show that it provides a quantitative measure the strength of proximity constraints in genome evolution and serves as a better predictor of functional links among genes than more naïve methods. It also sheds insight into the relationship between evolutionary development and the functional selection.

## Results

We applied our computational pipeline to 127 microbial genomes which were obtained from the NCBI website (2/2004). Pairwise genome comparisons of translated open reading frames were performed using BLASTP [17] and putative orthologs between genomes were identified as reciprocal best hits (see Methods). Conservation scores *C*_*u *_and *C*_*d *_were assigned to each gene (see Methods). Intuitively, *C*_*u *_measures the strength of the conservation between the gene under consideration and its upstream neighboring genes while *C*_*d *_does the same with its downstream neighboring genes on the chromosome. The higher the *C*_*u *_or *C*_*d *_of a gene is, the more significant its conservation with its upstream or downstream neighbors. Conserved gene clusters were then detected using a conservation score cutoff (see Methods section). The complete results of our method on all microbial genomes included can be accessed at [18]. Results using more than 200 genomes available now are being incorporated into the database.

### Accounting for genome phylogeny can correct for databases bias

**Figure 1:**
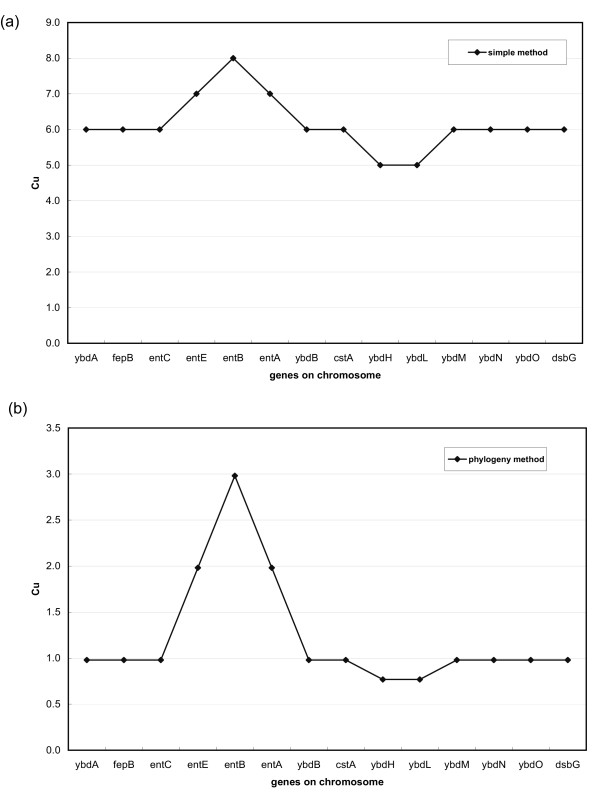
**Improvement of signal-to-noise ratio of the phylogenetic method over the simple method**. The upper conservation scores (C_u_) profiles for the genomic region surrounding *ent *operons in *Escherichia coli *are shown. (a) the simple method; (b) the phylogenetic method.

During evolution, genomes undergo frequent changes such as the rearrangement and exchange of genes. For those organisms that have diverged recently, such changes have had little time to occur so that vertically inherited synteny cannot be distinguished from functionally induced proximity information. The false positive rate could therefore drastically increase as more and more genomes from the same taxonomic group are included in the comparison. Early methods ignored the phylogenetic relationship between genomes and assumed an independence between them, which could blur the distinction between gene clusters truly constrained by selection pressures and those that merely reflect vertical inheritance. The phylogenetic method we describe here is designed to integrate genome phylogenetic information into consideration and alleviate the common problem of overestimation. For illustration we devised an experiment and compared the performance of our method with the more naïve method where each genome is treated equally, which we refer to hereafter as "simple counting method". First, we chose *E. coli *K12 as the query genome and a group of 6 reference genomes including 3 closely related ones (*E. coli *O157H7, *E. coli *CFT073 and *E. coli *O157H7 EDL933) and 3 distantly related ones (*Mycobacterium tuberculosis *CDC1551, *Staphylococcus aureus *Mu50 and *Bacillus subtilis*). We then examined the genomic region of the *entCEBA *operon in *E. coli *K12 [19] and used two methods to calculate the upstream conservation score (C_u_) as defined in the methods section. In the simple counting method, when a gene and its upstream gene are also neighbors in a reference genome, the score for that gene will be incremented by 1. Figure 1a and 1b show the upstream conservation score profiles obtained from the simple counting method and our phylogenetic method respectively. In Figure 1a, the scores of the *entCEBA *operon genes barely stands out from those of the flanking genes while in Figure 1b there is a clear peak corresponding to the *entCEBA *operon. The signal to noise ratio, as estimated from the peak value and the base line of the C_u _profile, is about 3:1 for the phylogenetic method (Figure 1b) but only about 1.3:1 for the simple method (Figure 1a).

**Figure 2:**
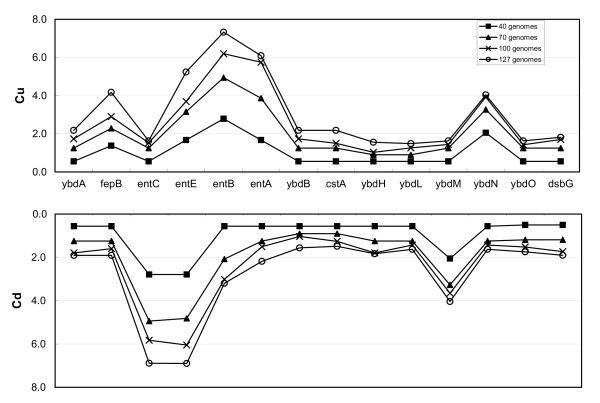
Conservation score (C_u_ and Cd) profiles for the *entCEBA *genomic region using increasing number of reference genomes.

The signal to noise ratio increases as more genomes are added to the reference set. The *C*_*u *_and *C*_*d *_profiles are shown in Figure 2 for the same genomic region around the *entCEBA *operon as we progressively include more genomes in the reference set. While the scores of the genes in the operon increase as more reference genomes are included, the conservation scores of the flanking genes increase much more slowly (Figure 2). Notice that *C*_*u *_for *entC *and *C*_*d *_for *entA *do not increase as much as other *ent *genes since they are the boundary genes of the *ent *operon. Figure 2 shows that the phylogenetic method provides an improved framework for detecting conserved gene clusters as the number of genomes in the database increases.

### Conserved gene clusters inferred from orthology or similarity

**Figure 3:**
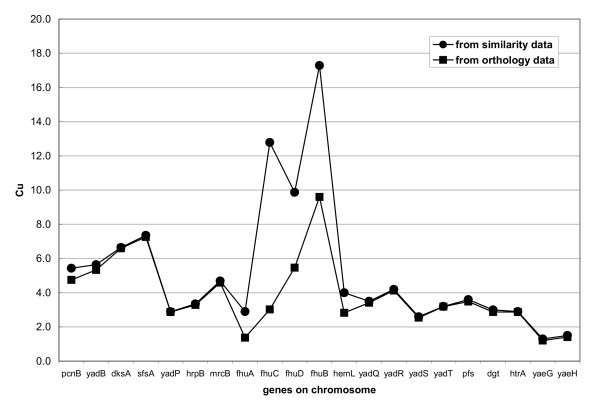
Upstream conservation score calculated from orthology and similarity data for a genomic region surrounding *fhuABCD *operon in *Escherichia coli*.

It is known that using reciprocal best BLAST hits is a conservative method of identifying putative orthologous genes between two genomes. The advantage of using this conservative method is that any pairs identified are very likely to be true orthologs and share similar molecular and physiological function. However, for genes that have undergone fast evolution and for paralogous gene groups, which are quite common in bacterial genomes, the reciprocal relationships are usually obscured [20]. As a result, significantly conserved clusters or parts of a cluster may be missed if pairwise reciprocal connections are used. This is illustrated in Figure 3, which shows the upstream conservation score (C_u_) profiles for a genomic region containing the *fhu *operon [21] of *E. coli*. Circles are calculated from the similarity data (BLAST E-value < 1E-5) and the squares are from the orthology data. For most genes where the orthology relationship is clear, scores calculated from the similarity data are equal to those from the orthology data (Figure 3). For those genes with no clear orthologs in other genomes, scores from similarity data are higher than those from orthology data. For instance, the *C*_*u *_of the *fhuC *gene from similarity data exceeds the threshold (5.0 used in this paper), but its *C*_*u *_from the orthology data does not. This is because *fhuA*, the immediately upstream gene of *fhuC*, belongs to a paralogous gene family with 9 members in *E. coli *and its reciprocal best hit cannot be found in other genomes. As a result, the gene pair (*fhuA*, *fhuC*) is not identified as a widely conserved orthologous pair. On the other hand, only using the similarity data may introduce a higher false positive rate in making predictions about functional dependence, as discussed below.

### Application in operon prediction

To test the predictive value of our method for inferring operons, we took a compiled list of *E. coli *operons from the RegulonDB [22]. A total of 345 operons with multiple genes were extracted from the RegulonDB dataset. Genes that are not in the RegulonDB set and have intergenic regions of larger than 100 nucleotides on both sides comprise the negative set (1342 genes). Using different cutoffs, we calculated the sensitivity (Sn) and the specificity (Sp) by:

Sn=TPTP+FN,Sp=TNFP+TN
 MathType@MTEF@5@5@+=feaafeart1ev1aaatCvAUfKttLearuWrP9MDH5MBPbIqV92AaeXatLxBI9gBaebbnrfifHhDYfgasaacH8akY=wiFfYdH8Gipec8Eeeu0xXdbba9frFj0=OqFfea0dXdd9vqai=hGuQ8kuc9pgc9s8qqaq=dirpe0xb9q8qiLsFr0=vr0=vr0dc8meaabaqaciaacaGaaeqabaqabeGadaaakeaacqWGtbWucqWGUbGBcqGH9aqpdaWcaaqaaiabdsfaujabdcfaqbqaaiabdsfaujabdcfaqjabgUcaRiabdAeagjabd6eaobaacqGGSaalcqWGtbWucqWGWbaCcqGH9aqpdaWcaaqaaiabdsfaujabd6eaobqaaiabdAeagjabdcfaqjabgUcaRiabdsfaujabd6eaobaaaaa@447F@

where TP and FP are the number of genes that are correctly or wrongly predicted to be in operons, and TN and FN are the number of genes that are correctly or wrongly predicted not to be in operons. Notice that genes in operons must reside on the same strand while genes in clusters detected by our system may come from both strands. Nevertheless, the high percentage of conserved clusters that overlap with operon dataset suggests our ability to identify clusters that are conserved due to the transcriptional selection pressure and demonstrates the potential use of the system in the task of operon prediction.

**Figure 4:**
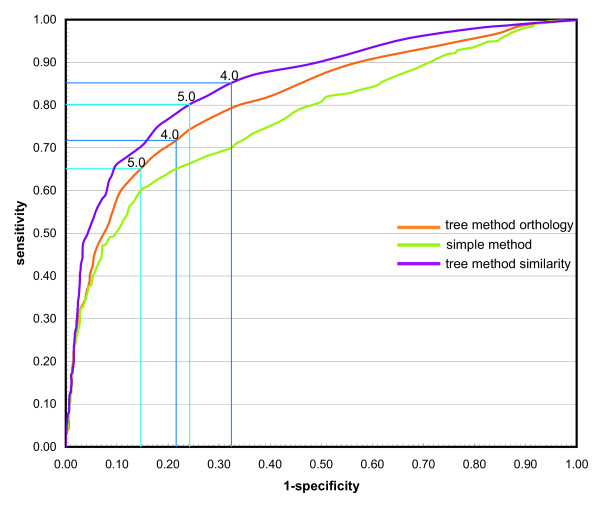
**Receiving operating characteristic (ROC) curves of different methods on the RegulonDB operon dataset**. Curves are color-coded for different methods. Points using cutoffs of 4.0 and 5.0 for our method are highlighted on the curve.

Figure 4 compares the performance of the two methods on the operon dataset by showing the receiver operating characteristic (ROC) curves. Our method using either orthology data or similarity data consistently outperforms the simple counting method, which treats each observation with equal weight (Figure 4). As a result, when using 5.0 (P-value < 2.4E-4, default cutoff) as the threshold for making operon predictions using orthology data, our method gives 65% sensitivity and 85% specificity in *E. coli *(Figure 4). For the same cutoff value (e.g., 4.0 or 5.0), the results using similarity data give better sensitivity but worse specificity than the results using orthology data (Figure 4). Higher specificity is achieved when a larger cutoff is used. For instance, when a reported cluster has a score (based on orthology) larger than 6.0 (P-value < 1.7E-4), there is an 89% chance that this cluster corresponds to an operon.

### Statistics of conserved gene clusters across genomes

Table 1 shows the statistics of conserved gene clusters in a number of microbial genomes where we have applied our method. Microbial genomes appear to have a highly specialized distribution of conserved clusters (Table 1). It appears that in general 10–40% of all the genes in a genome lie in conserved gene clusters (based on orthology data). Among the identified clusters, there are very few that are widely conserved across all species or conserved with invariant gene content (Y.Z. et al, unpublished results). Although it appears that in several cases the high content of conserved gene clusters occurs in relatively small genomes (Table 1), the overall correlation between the genome size and the proportion of conserved gene clusters is not significant (r^2^≈0.1).

**Table 1:** Statistics of conserved gene clusters in a number of microorganisms

Genome	Total genes	Total genes in clusters	Total detected clusters	Percentage	Average cluster size
Chlamydophila pneumoniae J138	1070	209	58	0.20	3.6
Mycobacterium tuberculosis CDC1551	4187	547	161	0.13	3.4
Sinorhizobium meliloti	6205	841	230	0.14	3.7
Clostridium acetobutylicum	3672	707	166	0.19	4.3
Mycobacterium tuberculosis H37Rv	3918	543	156	0.14	3.5
Staphylococcus aureus Mu50	2748	757	174	0.28	4.4
Aeropyrum pernix	2694	160	44	0.06	3.6
Clostridium perfringens	2723	633	147	0.23	4.3
Mycoplasma genitalium	480	153	40	0.32	3.8
Agrobacterium tumefaciens C58	5301	805	221	0.15	3.6
Deinococcus radiodurans	3102	389	117	0.13	3.3
Mycoplasma pneumoniae	688	167	47	0.24	3.6
Streptococcus pneumoniae R6	2043	546	151	0.27	3.6
Agrobacterium tumefaciens C58 UWash	5402	832	224	0.15	3.7
Escherichia coli K12	4289	1313	287	0.31	4.6
Mycoplasma pulmonis	782	168	52	0.21	3.2
Streptococcus pneumoniae TIGR4	2094	534	147	0.26	3.6
Escherichia coli O157H7	5361	1327	288	0.25	4.6
Neisseria meningitidis MC58	2025	457	129	0.23	3.5
Streptococcus pyogenes	1696	501	136	0.30	3.7
Aquifex aeolicus	1553	178	57	0.11	3.1
Sulfolobus solfataricus	2977	244	65	0.08	3.8
Archaeoglobus fulgidus	2407	250	73	0.10	3.4
Nostoc sp	6129	284	88	0.05	3.2
Sulfolobus tokodaii	2826	242	65	0.09	3.7
Bacillus halodurans	4066	952	219	0.23	4.3
Borrelia burgdorferi	1709	214	57	0.13	3.8

### Case studies of conserved gene clusters

Conserved gene clusters, once identified, can be used to make functional predictions for the genes within them, and to hypothesize interactions between their products. For example, in the genome of *Mycobacterium tuberculosis *CDC1551, we found a four-gene cluster encoding putative homologs of (1) *mraZ *(MT2224), (2) *mraW/yabC *(MT2223), (3) *ftsL/mraR *(MT2222), and (4) *ftsI/pbpB *(MT2221). This cluster is widely conserved among both gram-positive and gram-negative bacteria, and in *E. coli *these four genes comprise the start of a known operon of cell division and cell envelope genes [23]. FtsI and FtsL are known to be essential for the assembly of the cell division septum in *E. coli*. MraW has been shown to be a methyltransferase whose substrates are localized to the cell envelope [24]. Furthermore, it has been shown that lack of S-adenosylmethionine (SAM) leads to a cell division defect in *E. coli*, with one possible explanation being that SAM serves as a methyl donor in a required methylation event [25]. The location of *mraW *within this cluster suggests it may encode the methylase involved in such an event, possibly modifying the FtsL and/or FtsI proteins. Indeed, there is experimental data to suggest this [23], although it has not yet been conclusively shown.

**Figure 5:**
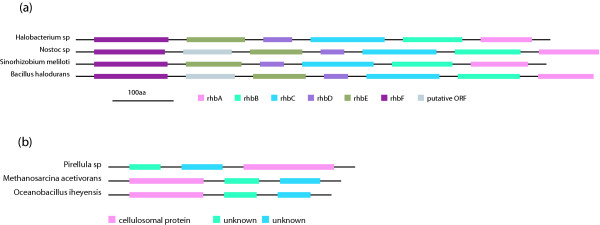
Examples of gene clusters that are conserved in only a few genomes.

In addition to conserved gene clusters that are widely distributed in many genomes, we also find statistically significant conserved gene clusters that are present in only a few species. Instead of the number of genomes, the large evolutionary span across genomes contributes to the significance of the conservation. Due to their limited occurrence, these clusters are difficult to find in the database using conventional methods. Figure 5 gives two intriguing examples: one is conserved in 4 genomes (Figure 5a) and the other is conserved in only 3 genomes (Figure 5b). In both examples the large evolutionary span is reflected by the fact that they are conserved in both bacterial and archaeal species.

The gene cluster in Figure 5a was originally found and characterized in *Sinorhizobium meliloti *and was shown to synthesize a special type of siderophore, rhizobactin 1021, involved in iron uptake [26]. It is largely conserved in another three genomes: one is an archaea, *Halobacterium sp.*, and the other two are bacteria, *Nostoc sp*. and *Bacillus halodurans*. Notice that this cluster is absent in other Bacilli species (*Bacillus subtillis*, etc.) despite their closeness to *Bacillus halodurans*. On the other hand, *Bacillus subtilis *is known to possess the *dhb* operon responsible for synthesizing a different type of siderophore, 2,3-dihydroxybenzoate [27], and this operon is absent in *Bacillus halodurans*. Figure 5a suggests that the other three microorganisms may possess the ability to synthesize rhizobactin 1021 or a siderophore with a similar structure, reflecting a requirement for iron in the life cycles of these species.

Figure 5b depicts a three gene cluster that is conserved in *Pirelulla sp.*, *Methanosarcina acetivorans *and *Oceanobacillus iheynesis*. One of the three genes is annotated as a cellulosomal protein. A BLAST search reveals its similarity to *cotH*, which has been characterized in *Bacillus subtilis *and is essential for spore coat assembly [28]. The other two are conserved hypothetical genes in Genbank with no functional information although they are both predicted to be transmembrane proteins (by TMpred). The conservation in Figure 5b suggests the two unknown genes are functionally related to cotH. We note that these conserved gene clusters that appear in just a few genomes with a large evolutionary span could be instances of horizontal transfer.

### Functional enrichment in conserved gene clusters

If conserved gene proximity indeed implies relatedness, we expect to see functional enrichment in the list of gene clusters found by our method. We used the 18 functional codes of COG [29] as a crude measure of this, counting gene pairs in which both members belong to the same COG category. For 39 genomes examined, we determined the fraction of gene pairs found by our method (*m*/*n*), then performed a one-tailed Fisher's exact test to determine the probability of observing at least *m *gene pairs with the same functional code among *n *pairs selected randomly from all gene pairs in the genome. Gene pairs including at least one gene with no functional information (*i.e.*, having no assigned category, or designated as general function or unknown function) were excluded from the analysis.

A partial list of the results of this analysis is shown in Table 2, with the fraction of enriched clusters and the associated P-values in columns 2 and 3, respectively. In all cases, these P-values are extremely significant (*P *<< 0.01), indicating that the gene pairs obtained by our method are much more likely to be functionally related than expected by chance.

**Table 2:** Statistics of functional enrichment

	**Phylogeny Method**		**Counting Method**		
		
**Organism**	**Fraction**	**P-value**	**Fraction**	**P-value**	**P-value ratio**^**a**^
Aeropyrum pernix	39/45	6.33E-12	40/46	2.21E-12	0.349
*Aquifex aeolicus*	80/107	1.23E-32	81/111	8.71E-32	7.08
*Bacillus subtilis*	329/503	1.86E-44	363/601	4.10E-38	2.20E+06
*Buchnera sp.*	167/246	1.28E-31	181/307	1.90E-23	1.48E+08
*Chlamydia trachomatis*	95/132	3.94E-24	123/295	1.88E-03	4.77E+20
*Chlamydophia pneumoniae*	104/137	5.03E-30	135/327	4.29E-03	8.53E+26
*Deinococcus radiodurans*	173/221	4.07E-57	167/218	5.56E-52	1.37E+05
*Escherichia coli K12*	439/750	8.92E-48	606/1553	0.19	2.13E+46
*Halobacterium sp.*	127/150	7.60E-38	125/145	6.27E-39	0.0825
*Helicobacter pylori 26695*	120/151	1.11E-37	122/158	4.92E-36	0.443
*Lactococcus lactis*	182/277	2.79E-28	196/319	4.19E-25	1500
*Methanococcus janaschii*	68/83	5.82E-22	64/79	5.91E-20	102
*Mycobacterium tuberculosis*	203/293	1.05E-42	226/347	1.09E-41	10.4
*Mycoplasma pulmonis*	75/98	3.61E-11	77/103	1.44E-10	3.99
*Neisseria meningitidis MC58*	150/222	1.25E-51	156/241	9.14E-51	7.31

^a ^Ratio is P-value for functional enrichment by the phylogeny method divided by P-value for functional enrichment by the counting method [i.e., (column 3) / (column 5)].

Using the same methodology, we divided the gene pairs into colinear (on the same strand) and divergent (on opposite strands) pairs to see if the bulk of functionally enriched pairs were of one type or the other. The P-values for colinear pairs were similar to those for all pairs, suggesting the vast majority of gene pairs captured by our method might be operons (data not shown). On the other hand, there are only limited numbers of divergent pairs and the P-values for divergent pairs are often insignificant (data not shown).

Functional enrichment analysis has been also applied to the results from the simple counting method, using a cutoff of 10 organisms (of 127 total). The results are shown in Table 3, columns 4 and 5. Although there is no equivalency between them (one is an absolute number, whereas the other is a log-odds score), the number of gene pairs captured by the two methods with their respective cutoffs were similar for many of the genomes examined (Table 3). In most cases, the simple counting method also yielded significantly functionally enriched gene pairs, as indicated by the P-values in column 5. For most genomes, the P-values of the simple counting method are much smaller than those from the phylogeny method (column 6 of Table 2). This is especially true for those genomes which have many close relatives in the database, *e.g.*, *E. coli *and other Enterobacteriaceae. In those cases, we also observe a large increase in the number of pairs obtained by the simple counting method. We expect this increase to consist largely of functionally unrelated false positives that are conserved because of close phylogenetic distance.

We recognize the limitations of using COG codes to capture functional relationships, and our results will certainly include both false positives and exclude false negatives. For example, consider a functionally related gene pair consisting of a transcriptional regulator and the gene it regulates. These genes would likely be assigned different COG codes, so such a functional relationship would not be captured by this analysis. Therefore, to independently verify our results with COG, we performed a similar analysis using KEGG pathway information in *E. coli*, the organism with the largest number of KEGG pathway assignments. The overrepresentation statistics for *E. coli *using KEGG are similar to our results using COG and thus support the above discussion (data not shown).

## Discussion

There is a general problem when comparing sequenced genomes because the samples of such genomes presently available are not uniformly distributed across the microbial kingdoms. Rather, several groups of related bacteria, such as the Enterobacteriaceae, are over-represented. This means that the significance of a feature found in organisms from this group must be interpreted with caution because it may be conserved by simple lineage effects rather than by functional selection. Most papers dealing with this problem either ignore it or simply remove the multiple members of the family and settle for a single representative example. Both methods necessarily lead to inaccuracies in estimating significance. In this paper, we have attempted to overcome this problem by using a method that takes into account the phylogenies of the individual members of related families. We show that it results in a reduction in the false positive rate over the simple counting method. We have not attempted to compare it to the selective sampling method, where one organism is used to represent a phylum, because given the wide variability usually observed within a phylum, the results from that approach will vary widely depending upon which genome is selected as the exemplar.

We have applied our new phylogenetically informed method to the problem of detecting conserved gene clusters. Such clusters are generally believed to reflect conservation of biological function in that often the gene products from the various genes in the cluster are involved in closely-related pathways. This may include the traditional operons known to encode the biosynthetic pathways of intermediary metabolism, or they may reflect the fact that enzymes responsible for post-translational modification will sometimes affect neighboring gene products. Other functional connections may also be found in these clusters. This can be a powerful tool in making predictions about gene products that might otherwise be recalcitrant to direct similarity analysis. Examples here include the restriction-modification enzymes where the DNA methyltransferases often show reasonable degrees of similarity that enable them to be identified, whereas the genes for the restriction enzymes evolve rapidly and usually cannot be identified on the basis of sequence similarity. Nevertheless, the genes are usually clustered. In addition, the current methodology is not necessarily restricted to the conserved synteny between genes. It may be applied to conserved synteny of other functional elements in genomes such as *cis*-elements, riboswitches, *etc*.

In our current methodology we have made several simplifying assumptions of which the most important might be to ignore horizontal gene transfer. While this is not a problem if an entire cluster is transferred horizontally, it does become a problem for those "hitchhiking" genes that may be transferred with the cluster. Nevertheless, the results we report seem promising and we do not view this as being a serious limitation at the present time.

The mode of phylogenetic inference is an important consideration in the type of analysis presented here. The basis for the tree underlying the analysis is not restricted to shared gene content, as we have employed here, but could alternatively be based on 16S RNA sequences, gene content, gene order, genome statistics, or coding sequences. Our current method is oblivious to "local" changes such as rearrangements inside the cluster, or differential selection within individual coding sequences. For example, it is possible that genes in the cluster might have different gene trees from each other. The choice of the best phylogenetic methodology in this context is an important follow-up and we feel that the final solution will combine the benefits of multiple methodologies in a single system.

## Conclusion

The methodology and results that we present here should be generally useful in any situation where the functional significance of conserved genes or clusters is being investigated, for example, as a way of cross-validating co-expressed genes inferred from many microarray experiments or as a starting point for the assembly of gene networks. As more and more genomes are sequenced, the approach described here should be generally applicable and it will scale well computationally.

Of particular significance in this paper has been our finding that many of the clusters we have observed contain unknown genes with no biological function currently assigned. In these cases it seems reasonable to hypothesize that the product of the unknown gene has a function closely related to the functions of the known genes. Such a function may be a key enzymatic step in a biosynthetic pathway, a key regulatory function or an important post-translational modification. We have assembled a database of conserved gene clusters, called GeneChords [18], with a simple user interface to permit its rapid query. This will be described in detail in a separate publication.

## Methods

Once a genome is chosen for analysis, our algorithm consists of the following steps:

a) For each gene pair (as defined below) in the genome, a tree-based conservation score is computed;

b) For each gene in the genome, two neighbourhood conservation scores are computed, measuring the strength of proximity constraint of the gene with its upstream or downstream neighboring genes;

c) Adjacent genes with conservation scores between them exceeding some threshold are joined into conserved clusters.

The details are described in the following sections.

### Definition of conserved neighboring gene pairs

For simplicity let us consider the problem of identification of a conserved gene pair, the building block of our gene clusters. We define a gene pair as two genes with no more than *k *open reading frames separating them along the chromosome (*k *= 1 in this paper). In contrast to the operon identification procedure [12], the two genes in a pair do not need to reside on the same strand of the chromosome. When orthologs of a gene pair form a pair in other genomes, the pair is considered conserved. Orthologous genes are often detected as reciprocal best hits (BLAST E-value < 1E-5) between the two genomes by sequence comparison software, such as BLAST [17]. However, evolutionary events such as gene duplication followed by diversification could obscure the reciprocal relationship, resulting in relatively high false negative rates in the identification of orthologous genes. To relieve this concern, we loosen the criteria to include genes with high similarity (BLAST E-value ≤ 1E-5) (see Results). However, unless specifically pointed out, the results presented are calculated on the basis of orthology data.

### Computation of conservation scores using phylogenetic information

We assign a score to a conserved gene pair by computing the probability a particular pattern of conservation is observed in analyzed genomes based on a stochastic model of evolution. Before introducing the detailed implementation, we first lay out the theoretical foundation of the method and point out our assumptions.

**Figure 6:**
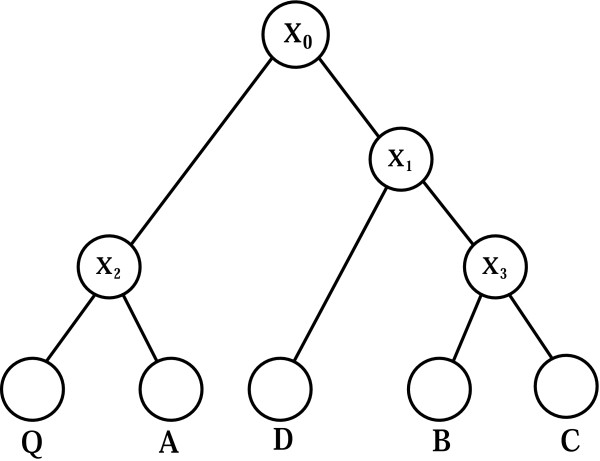
A simple genome phylogenetic tree.

As an example, consider the rooted bifurcating phylogenetic tree shown in Figure 6. The leaf nodes Q, A, B, C and D represent extant genomes, and internal nodes X_0_, X_1_, X_2 _and X_3 _are inferred ancestor genomes. Let us assume a gene pair in Q is conserved in A, B and C but not D. We model evolution as a stochastic process represented as a probabilistic graphical model in the form of a tree [30]. We associate a binary random variable with each node in the tree, assigning it a value 1 if the specific gene pair is conserved in the genome and 0 if absent. The values of leaves in the tree are determined by our initial gene cluster identification procedure.

We assume that the probability of a genome acquiring a gene cluster given its absence in the ancestor is negligible, *i.e.*, P(child = 1|parent = 0) = 0. This is a simplifying assumption which considers the predominance of vertical inheritance and omits the negligible probability of independent formation of identical clusters. Under this assumption the most recent common ancestor of all the leaves that are assigned to 1 is also set to 1. Accordingly, in the Figure 6 tree model, X_0 _is set to 1. We compute the significance of the conservation based on the probability of observing the specific gene cluster given that the closest common ancestor has it, that is,

*P*(*conservation*) = *P*(*Q *= 1, *A *= 1, *B *= 1, *C *= 1, *D *= 0|*X*_0 _= 1)

In our initial model we do not take into account the genomes that do not possess the cluster, although it is not difficult to do with the appropriate assumption on the conditional probability of loss of a cluster in a descendant of an organism that has it. Thus a leaf node D that lacks the cluster is dropped from further calculation. Now we have

*P*(*conservation*) = *P*(*Q *= 1, *A *= 1, *B *= 1, *C *= 1|*X*_0 _= 1)

The tree in Figure 6 can also be interpreted as a Bayesian network in a tree form [31,32]. In particular, the vertical inheritance along any path in the tree is a generative probabilistic process, and the probability that a child inherits a gene pair is only dependent on its immediate evolutionary ancestor. More specifically, we associate a conditional probability table with each edge of the tree enumerating the probability of the presence or absence of a gene pair in a genome given the state of its immediate ancestor. According to the tree model, we have

P(Q=1,A=1,B=1,C=1|X0=1)=∑X2P(Q=1,A=1|X2)⋅P(X2)∑X3P(B=1,C=1|X3)⋅P(X3)
 MathType@MTEF@5@5@+=feaafiart1ev1aaatCvAUfKttLearuWrP9MDH5MBPbIqV92AaeXatLxBI9gBaebbnrfifHhDYfgasaacH8akY=wiFfYdH8Gipec8Eeeu0xXdbba9frFj0=OqFfea0dXdd9vqai=hGuQ8kuc9pgc9s8qqaq=dirpe0xb9q8qiLsFr0=vr0=vr0dc8meaabaqaciaacaGaaeqabaqabeGadaaakqaabeqaaiabdcfaqjabcIcaOiabdgfarjabg2da9iabigdaXiabcYcaSiabdgeabjabg2da9iabigdaXiabcYcaSiabdkeacjabg2da9iabigdaXiabcYcaSiabdoeadjabg2da9iabigdaXiabcYha8jabdIfaynaaBaaaleaacqaIWaamaeqaaOGaeyypa0JaeGymaeJaeiykaKIaeyypa0dabaWaaabuaeaacqWGqbaucqGGOaakcqWGrbqucqGH9aqpcqaIXaqmcqGGSaalcqWGbbqqcqGH9aqpcqaIXaqmcqGG8baFcqWGybawdaWgaaWcbaGaeGOmaidabeaakiabcMcaPiabgwSixlabdcfaqjabcIcaOiabdIfaynaaBaaaleaacqaIYaGmaeqaaOGaeiykaKYaaabuaeaacqWGqbaucqGGOaakcqWGcbGqcqGH9aqpcqaIXaqmcqGGSaalcqWGdbWqcqGH9aqpcqaIXaqmcqGG8baFcqWGybawdaWgaaWcbaGaeG4mamdabeaakiabcMcaPiabgwSixlabdcfaqjabcIcaOiabdIfaynaaBaaaleaacqaIZaWmaeqaaOGaeiykaKcaleaacqWGybawdaWgaaadbaGaeG4mamdabeaaaSqab0GaeyyeIuoaaSqaaiabdIfaynaaBaaameaacqaIYaGmaeqaaaWcbeqdcqGHris5aaaaaa@78B1@

Assuming independence between the siblings, the above can be rewritten as

P(Q=1,A=1,B=1,C=1|X0=1)=∑X2P(Q=1|X2)⋅P(A=1|X2)⋅P(X2)∑X3P(B=1|X3)⋅P(C=1|X3)⋅P(X3)
 MathType@MTEF@5@5@+=feaafeart1ev1aaatCvAUfKttLearuWrP9MDH5MBPbIqV92AaeXatLxBI9gBaebbnrfifHhDYfgasaacH8akY=wiFfYdH8Gipec8Eeeu0xXdbba9frFj0=OqFfea0dXdd9vqai=hGuQ8kuc9pgc9s8qqaq=dirpe0xb9q8qiLsFr0=vr0=vr0dc8meaabaqaciaacaGaaeqabaqabeGadaaakqaabeqaaiabdcfaqjabcIcaOiabdgfarjabg2da9iabigdaXiabcYcaSiabdgeabjabg2da9iabigdaXiabcYcaSiabdkeacjabg2da9iabigdaXiabcYcaSiabdoeadjabg2da9iabigdaXiabcYha8jabdIfaynaaBaaaleaacqaIWaamaeqaaOGaeyypa0JaeGymaeJaeiykaKIaeyypa0dabaWaaabuaeaacqWGqbaucqGGOaakcqWGrbqucqGH9aqpcqaIXaqmcqGG8baFcqWGybawdaWgaaWcbaGaeGOmaidabeaakiabcMcaPiabgwSixlabdcfaqjabcIcaOiabdgeabjabg2da9iabigdaXiabcYha8jabdIfaynaaBaaaleaacqaIYaGmaeqaaOGaeiykaKIaeyyXICTaemiuaaLaeiikaGIaemiwaG1aaSbaaSqaaiabikdaYaqabaGccqGGPaqkdaaeqbqaaiabdcfaqjabcIcaOiabdkeacjabg2da9iabigdaXiabcYha8jabdIfaynaaBaaaleaacqaIZaWmaeqaaOGaeiykaKIaeyyXICTaemiuaaLaeiikaGIaem4qamKaeyypa0JaeGymaeJaeiiFaWNaemiwaG1aaSbaaSqaaiabiodaZaqabaGccqGGPaqkcqGHflY1cqWGqbaucqGGOaakcqWGybawdaWgaaWcbaGaeG4mamdabeaakiabcMcaPaWcbaGaemiwaG1aaSbaaWqaaiabiodaZaqabaaaleqaniabggHiLdaaleaacqWGybawdaWgaaadbaGaeGOmaidabeaaaSqab0GaeyyeIuoaaaaa@88FE@

Note that this derivation is a simple generalization of the forward algorithm [30,32,33]. According to our vertical inheritance assumption, the probability for a child to have a gene pair is approximately zero if the immediate ancestor does not have the gene pair. After applying this assumption and evaluating the equation recursively, the above formula reduces to

*P*(*Q *= 1, *A *= 1, *B *= 1, *C *= 1|*X*_0 _= 1) = *P*(*Q *= 1|*X*_2 _= 1)·*P*(*A *= 1|*X*_2 _= 1)·*P*(*B *= 1|*X*_3 _= 1)·*P*(*C *= 1|*X*_3 _= 1)·*P*(*X*_2 _= 1|*X*_0 _= 1)·*P*(*X*_3 _= 1|*X*_0 _= 1)

More generally, for a gene pair found in a set of genomes and a given genome phylogeny tree T, the following simple relation holds

P(Q=1,A=1,B=1,...)=∏X,Y∈TP(X=1|Y=1),
 MathType@MTEF@5@5@+=feaafeart1ev1aaatCvAUfKttLearuWrP9MDH5MBPbIqV92AaeXatLxBI9gBaebbnrfifHhDYfgasaacH8akY=wiFfYdH8Gipec8Eeeu0xXdbba9frFj0=OqFfea0dXdd9vqai=hGuQ8kuc9pgc9s8qqaq=dirpe0xb9q8qiLsFr0=vr0=vr0dc8meaabaqaciaacaGaaeqabaqabeGadaaakeaacqWGqbaucqGGOaakcqWGrbqucqGH9aqpcqaIXaqmcqGGSaalcqWGbbqqcqGH9aqpcqaIXaqmcqGGSaalcqWGcbGqcqGH9aqpcqaIXaqmcqGGSaalcqGGUaGlcqGGUaGlcqGGUaGlcqGGPaqkcqGH9aqpdaqeqbqaaiabdcfaqjabcIcaOiabdIfayjabg2da9iabigdaXiabcYha8jabdMfazjabg2da9iabigdaXiabcMcaPaWcbaGaemiwaGLaeiilaWIaemywaKLaeyicI4SaemivaqfabeqdcqGHpis1aOGaeiilaWcaaa@52BD@

where Y is an immediate ancestor of X in T.

Taking the negative logarithm of both sides, we have

log⁡(P(Q=1,A=1,B=1,...))=∑X,Y∈Tlog⁡(P(X=1|Y=1))
 MathType@MTEF@5@5@+=feaafeart1ev1aaatCvAUfKttLearuWrP9MDH5MBPbIqV92AaeXatLxBI9gBaebbnrfifHhDYfgasaacH8akY=wiFfYdH8Gipec8Eeeu0xXdbba9frFj0=OqFfea0dXdd9vqai=hGuQ8kuc9pgc9s8qqaq=dirpe0xb9q8qiLsFr0=vr0=vr0dc8meaabaqaciaacaGaaeqabaqabeGadaaakeaacyGGSbaBcqGGVbWBcqGGNbWzcqGGOaakcqWGqbaucqGGOaakcqWGrbqucqGH9aqpcqaIXaqmcqGGSaalcqWGbbqqcqGH9aqpcqaIXaqmcqGGSaalcqWGcbGqcqGH9aqpcqaIXaqmcqGGSaalcqGGUaGlcqGGUaGlcqGGUaGlcqGGPaqkcqGGPaqkcqGH9aqpdaaeqbqaaiGbcYgaSjabc+gaVjabcEgaNjabcIcaOiabdcfaqjabcIcaOiabdIfayjabg2da9iabigdaXiabcYha8jabdMfazjabg2da9iabigdaXiabcMcaPiabcMcaPaWcbaGaemiwaGLaeiilaWIaemywaKLaeyicI4SaemivaqfabeqdcqGHris5aaaa@5D84@

Thus the probability of conservation of a gene cluster in a given tree is simply the sum of log conditional probabilities of all the branches on paths leading to the genomes where the gene pair is present.

We further assume that log(P(X = 1|Y = 1) is proportional to the length of the branch that connects X and its parent Y, that is, log(*P*(*X *= 1|*Y *= 1)) ~ d(*X,Y*). Thus,

log⁡(P(Q=1,A=1,B=1,...))∼∑X,Y∈Td(X,Y),
 MathType@MTEF@5@5@+=feaafeart1ev1aaatCvAUfKttLearuWrP9MDH5MBPbIqV92AaeXatLxBI9gBaebbnrfifHhDYfgasaacH8akY=wiFfYdH8Gipec8Eeeu0xXdbba9frFj0=OqFfea0dXdd9vqai=hGuQ8kuc9pgc9s8qqaq=dirpe0xb9q8qiLsFr0=vr0=vr0dc8meaabaqaciaacaGaaeqabaqabeGadaaakeaacyGGSbaBcqGGVbWBcqGGNbWzcqGGOaakcqWGqbaucqGGOaakcqWGrbqucqGH9aqpcqaIXaqmcqGGSaalcqWGbbqqcqGH9aqpcqaIXaqmcqGGSaalcqWGcbGqcqGH9aqpcqaIXaqmcqGGSaalcqGGUaGlcqGGUaGlcqGGUaGlcqGGPaqkcqGGPaqkcqWI8iIodaaeqbqaaiabdsgaKjabcIcaOiabdIfayjabcYcaSiabdMfazjabcMcaPaWcbaGaemiwaGLaeiilaWIaemywaKLaeyicI4SaemivaqfabeqdcqGHris5aOGaeiilaWcaaa@545D@

where Y is the parent of X. The summation of all the tree branches in the phylogenetic tree is proportional to the logarithm of the overall probability.

In our implementation, the genome phylogenetic tree is built by using the genome distance metric based on the shared gene content suggested by Snel and coworkers [34]. We first calculate the pairwise distance between genomes by *d *= -ln(*s*), where s = (number of shared orthologs)/(average of total gene numbers in two genomes). In this implementation, *Escherichia coli *and *Salmonella typhi*, have a distance of 0.35, while *Escherichia coli *and *Bacillus subtilis*, which are much more distantly related, have a larger distance of 1.18. The genome phylogenetic tree is then constructed from the pairwise distance matrix using the neighbor joining algorithm [35].

For each gene pair, a genome phylogenetic tree is built on the genomes that have the pair, the conservation score of the gene pair is the summation of all the branch lengths in the tree. Notice that another nice property of the score is that it is independent of the query genome.

### Detection of long conserved gene clusters

We now extend the methodology by using the gene pair conservation to detect longer conserved gene clusters. For the *g*th gene on the query chromosome, we estimate its upstream conservation score (*C_u_*) and downstream conservation score (*C_d_*) by:

Cu(g)=∑i=1k+1s(g−i,g)Cd(g)=∑i=1k+1s(g,g+i)
 MathType@MTEF@5@5@+=feaafeart1ev1aaatCvAUfKttLearuWrP9MDH5MBPbIqV92AaeXatLxBI9gBaebbnrfifHhDYfgasaacH8akY=wiFfYdH8Gipec8Eeeu0xXdbba9frFj0=OqFfea0dXdd9vqai=hGuQ8kuc9pgc9s8qqaq=dirpe0xb9q8qiLsFr0=vr0=vr0dc8meaabaqaciaacaGaaeqabaqabeGadaaakqaabeqaaiabdoeadnaaBaaaleaacqWG1bqDaeqaaOGaeiikaGIaem4zaCMaeiykaKIaeyypa0ZaaabCaeaacqWGZbWCcqGGOaakcqWGNbWzcqGHsislcqWGPbqAcqGGSaalcqWGNbWzcqGGPaqkaSqaaiabdMgaPjabg2da9iabigdaXaqaaiabdUgaRjabgUcaRiabigdaXaqdcqGHris5aaGcbaGaem4qam0aaSbaaSqaaiabdsgaKbqabaGccqGGOaakcqWGNbWzcqGGPaqkcqGH9aqpdaaeWbqaaiabdohaZjabcIcaOiabdEgaNjabcYcaSiabdEgaNjabgUcaRiabdMgaPjabcMcaPaWcbaGaemyAaKMaeyypa0JaeGymaedabaGaem4AaSMaey4kaSIaeGymaedaniabggHiLdaaaaa@5D93@

where each *s*(*g-i, g*) is the conservation score assigned to gene pair (*g-i, g*) calculated using the method above; *k *is the maximum number of intervening genes allowed in a conserved gene pair (*k* = 1 in this paper). As a result, each gene will be associated with two numerical scores, measuring the extent of conservation between itself and its upstream or downstream neighboring genes respectively.

The statistical significance of the conservation scores is inferred from a bootstrap simulation. For each genome, the null distribution is computed by calculating the conservation scores of the randomly shuffled genome. The P-value cutoff is set at about 1E-4 in this paper, which corresponds to a conservation score of about 5.0 for most genomes. Notice that P-values are related to genome size since genes in very small genomes may have higher chance of forming conserved gene clusters. For instance, conservation score of 5.0 corresponds to a larger P-value (3.5E-3) in a small genome *Mycoplasma genitalium *than in *E. coli *(2.4E-4).

For genes that are at the boundaries of the cluster, only one of the conservation scores will exceed the threshold, which provides a convenient way of detecting the boundaries. We detect the maximal conserved gene clusters by scanning the genomes sequentially. The gene with *C*_*d *_over the threshold, but not for its upstream genes, marks the start of a new cluster. The gene whose downstream genes have *C*_*u *_scores below the threshold mark the end of the cluster. All the genes between are considered as part of the cluster.

More sophisticated dynamic programming procedures, or single-linkage clustering algorithm to identify maximal conserved gene clusters are also possible.

The main program was written in C and used the LEDA library for the manipulation of trees. Scripts for generating genome BLAST data and for analyzing data were written in Perl. GeneChords was built with PostGreSQL. All scripts are available upon request from  the authors.

## Authors' contributions

YZ and SK devised the method. YZ carried out the implementation of the method and database. BPA carried out the functional enrichment analysis. All authors participated in interpreting the results and writing the manuscript. RJR and SK conceived the study. All authors read and approved the final manuscript.

## References

[B1] Overbeek R, Fonstein M, D'Souza M, Pusch GD, Maltsev N (1999). The use of gene clusters to infer functional coupling. Proc Natl Acad Sci U S A.

[B2] Yanai I, Derti A, DeLisi C (2001). Genes linked by fusion events are generally of the same functional category: a systematic analysis of 30 microbial genomes. Proc Natl Acad Sci U S A.

[B3] Wolf YI, Rogozin IB, Kondrashov AS, Koonin EV (2001). Genome alignment, evolution of prokaryotic genome organization, and prediction of gene function using genomic context. Genome Res.

[B4] Jacob F, Monod J (1961). Genetic regulatory mechanisms in the synthesis of proteins. J Mol Biol.

[B5] Zheng Y, Szustakowski JD, Fortnow L, Roberts RJ, Kasif S (2002). Computational identification of operons in microbial genomes. Genome Res.

[B6] Snel B, Bork P, Huynen MA (2002). The identification of functional modules from the genomic association of genes. Proc Natl Acad Sci U S A.

[B7] Snel B, Lehmann G, Bork P, Huynen MA (2000). STRING: a web-server to retrieve and display the repeatedly occurring neighbourhood of a gene. Nucleic Acids Res.

[B8] Lin LF, Posfai J, Roberts RJ, Kong H (2001). Comparative genomics of the restriction-modification systems in Helicobacter pylori. Proc Natl Acad Sci U S A.

[B9] Galperin MY, Nikolskaya AN, Koonin EV (2001). Novel domains of the prokaryotic two-component signal transduction systems. FEMS Microbiol Lett.

[B10] Yanai I, Mellor JC, DeLisi C (2002). Identifying functional links between genes using conserved chromosomal proximity. Trends Genet.

[B11] Bowers PM, Pellegrini M, Thompson MJ, Fierro J, Yeates TO, Eisenberg D (2004). Prolinks: a database of protein functional linkages derived from coevolution. Genome Biol.

[B12] Ermolaeva MD, White O, Salzberg SL (2001). Prediction of operons in microbial genomes. Nucleic Acids Res.

[B13] Kolesov G, Mewes HW, Frishman D (2001). SNAPping up functionally related genes based on context information: a colinearity-free approach. J Mol Biol.

[B14] Rogozin IB, Makarova KS, Murvai J, Czabarka E, Wolf YI, Tatusov RL, Szekely LA, Koonin EV (2002). Connected gene neighborhoods in prokaryotic genomes. Nucleic Acids Res.

[B15] Rogozin IB, Makarova KS, Wolf YI, Koonin EV (2004). Computational approaches for the analysis of gene neighbourhoods in prokaryotic genomes. Brief Bioinform.

[B16] Strong M, Mallick P, Pellegrini M, Thompson MJ, Eisenberg D (2003). Inference of protein function and protein linkages in Mycobacterium tuberculosis based on prokaryotic genome organization: a combined computational approach. Genome Biol.

[B17] Altschul SF, Madden TL, Schaffer AA, Zhang J, Zhang Z, Miller W, Lipman DJ (1997). Gapped BLAST and PSI-BLAST: a new generation of protein database search programs. Nucleic Acids Res.

[B18] GeneChords. http://genomics10.bu.edu/GeneChords.

[B19] Nahlik MS, Brickman TJ, Ozenberger BA, McIntosh MA (1989). Nucleotide sequence and transcriptional organization of the Escherichia coli enterobactin biosynthesis cistrons entB and entA. J Bacteriol.

[B20] Sonnhammer EL, Koonin EV (2002). Orthology, paralogy and proposed classification for paralog subtypes. Trends Genet.

[B21] Fecker L, Braun V (1983). Cloning and expression of the fhu genes involved in iron(III)-hydroxamate uptake by Escherichia coli. J Bacteriol.

[B22] Salgado H, Gama-Castro S, Martinez-Antonio A, Diaz-Peredo E, Sanchez-Solano F, Peralta-Gil M, Garcia-Alonso D, Jimenez-Jacinto V, Santos-Zavaleta A, Bonavides-Martinez C, Collado-Vides J (2004). RegulonDB (version 4.0): transcriptional regulation, operon organization and growth conditions in Escherichia coli K-12. Nucleic Acids Res.

[B23] Hara H, Yasuda S, Horiuchi K, Park JT (1997). A promoter for the first nine genes of the Escherichia coli mra cluster of cell division and cell envelope biosynthesis genes, including ftsI and ftsW. J Bacteriol.

[B24] Carrion M, Gomez MJ, Merchante-Schubert R, Dongarra S, Ayala JA (1999). mraW, an essential gene at the dcw cluster of Escherichia coli codes for a cytoplasmic protein with methyltransferase activity. Biochimie.

[B25] Newman EB, Budman LI, Chan EC, Greene RC, Lin RT, Woldringh CL, D'Ari R (1998). Lack of S-adenosylmethionine results in a cell division defect in Escherichia coli. J Bacteriol.

[B26] Lynch D, O'Brien J, Welch T, Clarke P, Cuiv PO, Crosa JH, O'Connell M (2001). Genetic organization of the region encoding regulation, biosynthesis, and transport of rhizobactin 1021, a siderophore produced by Sinorhizobium meliloti. J Bacteriol.

[B27] Hoffmann T, Schutz A, Brosius M, Volker A, Volker U, Bremer E (2002). High-salinity-induced iron limitation in Bacillus subtilis. J Bacteriol.

[B28] Naclerio G, Baccigalupi L, Zilhao R, De Felice M, Ricca E (1996). Bacillus subtilis spore coat assembly requires cotH gene expression. J Bacteriol.

[B29] Tatusov RL, Koonin EV, Lipman DJ (1997). A genomic perspective on protein families. Science.

[B30] Salzberg SL, Searls DB, Kasif S (1999). Computational methods in molecular biology.

[B31] Cai D, Delcher A, Kao B, Kasif S (2000). Modeling splice sites with Bayes networks. Bioinformatics.

[B32] Pearl J (1991). Probabilistic reasoning in intelligent systems: networks of plausible inference.

[B33] Durbin RESKAMG (1998). Biological sequence analysis.

[B34] Snel B, Bork P, Huynen MA (1999). Genome phylogeny based on gene content. Nat Genet.

[B35] Saitou N, Nei M (1987). The neighbor-joining method: a new method for reconstructing phylogenetic trees. Mol Biol Evol.

